# Richter transformation with marked plasmacytic differentiation, mimicking plasma cell neoplasm

**DOI:** 10.1002/jha2.376

**Published:** 2022-01-11

**Authors:** Shaoying Li, Jie Xu, C. Cameron Yin, Sergej Konoplev

**Affiliations:** ^1^ Department of Hematopathology The University of Texas MD Anderson Cancer Center Houston Texas USA

 
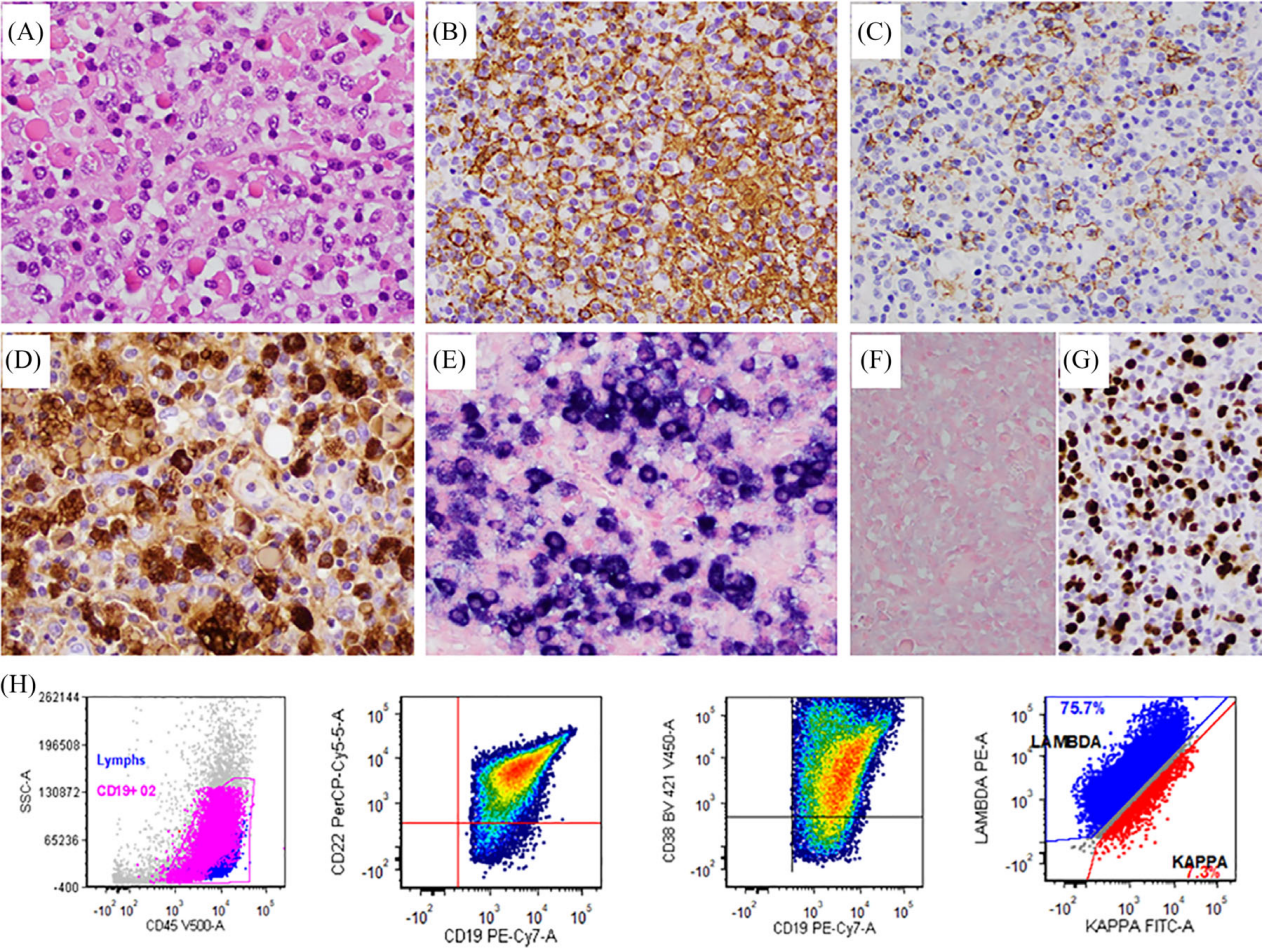


The patient is a 75‐year‐old man with a history of chronic lymphocytic leukemia (CLL), status post three courses of chemotherapy, and reached complete remission (CR). He presented with a right elbow mass. PET‐CT showed FDG‐avid nodular lesion in the soft tissue, most notably in the right upper elbow and left gluteal region, and also in the left lower lobe of the lung. The largest nodule in the right elbow measured 5.5 × 4.5 cm, which was biopsied. The biopsy showed diffuse infiltration of large neoplastic cells with round nuclei, fine chromatin, and prominent nucleoli. Admixed are many plasmacytoid lymphocytes and plasma cells, some showing abundant cytoplasmic Russell bodies (Mott cells) (**A**), suggestive of plasmacytic differentiation. Immunohistochemical stains showed that the large lymphoma cells were positive for CD20 (**B**), CD138 (subset, plasma cells, and some plasmacytoid lymphocytes, **C**), IgM (**D**), BCL2, BCL6, and MUM1, and negative for CD3, CD5, and IgG. By in‐situ hybridization study, the neoplastic cells demonstrated immunoglobulin lambda (**E**) light chain restriction and negative for kappa (**F**). Ki‐67 proliferation index was 40–50% (**G**). Lambda and IgM stains also highlighted the Russell bodies. Flow cytometry immunophenotype analysis detected an abnormal lambda restricted population of large B‐cells which were positive for CD19, CD20dim, CD22, CD38, CD79b, and negative for CD5, CD10, CD23, CD30, CD43, and CD200 (**H**). FISH showed no “MYC” rearrangements. The morphology and immunophenotype were diagnostic of diffuse large B cell lymphoma (DLBCL, Richter transformation) with marked plasmacytic differentiation and Mott cells. The patient was switched to blinatumomab and reached CR 5 months later, but relapsed after one year. He further received six cycles of R‐CHOP plus venetoclax and reached CR until now.

The most common form of CLL with Richter transformation is diffuse large B‐cell lymphoma, which occurs in 2–8% of CLL patients. Richter transformation with marked plasmacytic differentiation and Mott cells, as seen in this case, is exceedingly rare and often creates diagnostic challenges and easy to be misdiagnosed as plasma cell neoplasm. Thorough immunophenotype evaluations are helpful for a correct diagnosis. Expression of multiple B‐cell markers (CD19, CD20, CD22, CD79b), not bright CD38, and surface light chain restriction are features that support a diagnosis of DLBCL with marked plasmacytic differentiation, while lack of B‐cell markers and surface light chain expression and bright CD38 and diffuse CD138 favor a plasma cell neoplasm.

## AUTHOR CONTRIBUTIONS

Shaoying Li designed and performed the research and wrote the paper, Jie Xu, C. Cameron Yin and Sergej Konoplev wrote the paper.

